# Cigarette Smoke Amplifies Inflammatory Response and Atherosclerosis Progression Through Activation of the H1R-TLR2/4-COX2 Axis

**DOI:** 10.3389/fimmu.2015.00572

**Published:** 2015-11-09

**Authors:** Rajat S. Barua, Mukut Sharma, Kottarappat N. Dileepan

**Affiliations:** ^1^Division of Cardiovascular Medicine, Kansas City Veterans Affairs Medical Center, Kansas City, MO, USA; ^2^Research Service, Kansas City Veterans Affairs Medical Center, Kansas City, MO, USA; ^3^Department of Medicine, Division of Allergy, Clinical Immunology and Rheumatology, University of Kansas Medical Center, Kansas City, KS, USA

**Keywords:** cigarette smoking, infection, atherosclerosis, mast cells, lipopolysaccharide, histamine, cyclooxygenase-2, toll-like receptors 2 and 4

## Abstract

Emerging evidence suggests that infection and persistent inflammation are key players in the pathogenesis of atherosclerotic cardiovascular disease (CVD). Although it is well established that cigarette smoke (CS) promotes atherosclerotic CVD, very little is known about the potential impact of the collective effects of CS and intermittent or chronic subclinical infection on atherosclerosis. Our previous studies demonstrated that mast cell-derived histamine and lipopolysaccharide (LPS) synergistically enhance endothelial cell inflammatory response. We further noted that the synergy between histamine and LPS was due to reciprocal upregulation of histamine receptor and Toll-like receptor 4 (TLR4) expression and functions. These results suggest that the combined and persistent effects of mast cell mediators and bacterial agents on the vasculature are risk factors of CVD. Our recent data demonstrated that CS extract enhances histamine- and LPS-induced expression of cyclooxygenase-2 (COX-2) in endothelial cells, suggesting that CS and mast cell mediators may collectively amplify inflammatory response in the vessel wall. We hypothesize that CS enhances histamine-mediated upregulation of TLR2/TLR4 signaling in the endothelium and promotes progression of atherosclerosis. This article presents our perspective on the modulatory effects of CS and nicotine on the “histamine-TLR-COX-2 axis.”

## Introduction

Atherosclerosis (AS) is the underlying cause of several cardiovascular disease (CVD)-associated complications, including myocardial infarction (MI), heart failure, and stroke. Early stages of atherogenesis involve endothelial cell activation, persistent inflammatory response, and altered vascular homeostasis. The fact that the ever-changing milieu of endothelial cells alters cytokine profile, prostanoid homeostasis, and oxidative stress and attracts proinflammatory cells strengthens the notion that AS is an inflammatory disease.

It is recognized that both innate and adaptive immune systems contribute to the inflammatory processes in AS. In this respect, the role of T-lymphocytes, B-cells, macrophages, dendritic cells, natural killer cells, and neutrophils has been extensively studied. Furthermore, the presence of increased number of mast cells (1–4) and their influence in modulating toll-like receptors (TLRs) appear to be key contributors to the atherosclerotic process (5, 6). The mechanisms by which mast cell-mediated upregulation of innate immune system promote progression of AS remain obscure.

Toll-like receptors are expressed in a variety of cell types including endothelial cells (5). The presence of TLR2 and TLR4 in endothelial cells and their link to the COX-2 pathway add to the interaction between the innate immune system and AS. Our laboratory was the first to report the synergy between histamine and TLR2/TLR4 signaling that leads to overexpression of COX-2 and IL-6 in human coronary artery endothelial cells (HCAECs) (5–7). We demonstrated that the synergy between histamine and bacterial agents is due to reciprocal upregulation of the expression of histamine H1 receptor (H1R), TLR2, and TLR4. These findings strongly suggest that histamine can amplify endothelial inflammatory responses initiated by both Gram-positive and Gram-negative bacterial products.

Cigarette smoke (CS) is a major lifestyle-related risk factor for CVD. However, the impact of CS on intermittent or chronic subclinical infection-induced vascular inflammation and atherogenic pathobiology is unclear. It is well recognized that cigarette smoking increases the risk of microbial infection (8), and CS induces histamine release from mast cells (9). Thus, it is reasonable to postulate that cross-communication between CS, histamine, and bacterial products may lead to amplified inflammatory response. Here, we present a novel paradigm that CS promotes atherogenesis by modulating mast cell-mediated innate immune upregulation and by enhancing persistent vascular inflammation.

## Endothelial Cells and Early Events in Atherosclerosis

Endothelial cells constitute the initial site of vascular changes that evolve into life-threatening occlusive lesions. The endothelium covers the tunica intima of the vessel wall and is directly exposed to the circulating milieu and pathogens. Further into the wall, the tunica media contains most of the resident smooth muscle cells and the adventitia on the outside contains mast cells, nerve endings, and microvessels. Endothelial cell activation by blood-borne proinflammatory agents shifts the vascular tone and local vascular homeostasis which, in turn, increase the risk of accelerated development of atherosclerotic lesions (10).

Activation of endothelial cells also increases the expression of adhesion molecules (VCAM-1, ICAM-1, and E-selectin) (11) that recruit monocytes, T-cells, B-cells, dendritic cells, mast cell precursors, and neutrophils into the vascular wall where low-density lipoprotein (LDL)-laden monocytes mature into foam cells. Altered vessel wall environment promotes smooth muscle cell proliferation and causes their migration from the media to the intima where they synthesize matrix proteins including collagen, elastin, and proteoglycans. Immune cells in the developing plaque between endothelial cells and the adventitia release proinflammatory cytokines, matrix metalloproteinases (MMPs), and reactive oxidants, which ultimately lead to plaque rupture and occlusive event (12).

Cardiovascular disease risk factors including cigarette smoking, hypercholesterolemia, and oxidative stress cause early endothelial dysfunction leading to atherosclerotic changes. Endothelial dysfunction is indicated by decreased production of vasodilatory and homeostatic molecules such as nitric oxide (NO) and prostacyclin (prostaglandin I_2_, PGI_2_) or increased production of vasoconstrictive molecules such as endothelin-1 (ET-1) and thromboxane A_2_ (TXA_2_). Early endothelial dysfunction promotes the transformation of quiescent endothelial cells toward an active phenotype that involves host defense response (13).

## Role of Cyclooxygenase-2 and Prostanoids in Vascular Inflammation and Atherosclerosis

The COX isozymes in concert with specific isomerases modulate cellular homeostasis and vascular tone through the production of several prostanoids. COX-1 is constitutively expressed in most cells, whereas COX-2 expression is induced at sites of inflammation. COX-2 expression is upregulated in activated macrophages and endothelial cells, which makes it a target for the regulation of atherogenesis (7, 14). Although COX-2 expression is transient in acute inflammation, it can be prolonged or persistent in immune disorders (15).

Biological effects of COX products depend on their relative concentrations and receptor densities as well as the cell types and microenvironment. Among the prostanoids, PGI_2_ and TXA_2_ have received considerable attention because of their opposing effects in the vasculature (16). Specific threshold levels of their concentrations in systemic circulation and vascular tissues are prerequisite for vascular homeostasis (17). PGI_2_ functions as a potent vasodilator and inhibitor of leukocyte adhesion and platelet aggregation. Early depletion of PGI_2_ from endothelium promotes AS by enhancing lipid deposition in smooth muscle cells (18). In contrast, TXA_2_ is a potent inducer of vasoconstriction, platelet activation, and platelet adhesion. Therefore, a shift in the PGI_2_/TXA_2_ equilibrium may dictate the vascular tone.

Our previous work focused on the interaction between histamine and lipopolysaccharide (LPS) with regard to COX-2 expression in endothelial cells (6, 7). The preferential effect of histamine on the induction of COX-2 expression with resultant production of PGE_2_ and PGI_2_ and its lack of influence on COX-1 expression or TXA_2_ production support the concept of a distinct coupling of COX-2 with PGE_2_ and PGI_2_ synthases and that of COX-1 with TXA_2_ synthase. It is intriguing that, despite the coexistence of both COX-1 and COX-2 in HCAEC, histamine is able to segregate its influence on COX-2/PGE_2_/PGI_2_ pathway and not on COX-1/TXA_2_ pathway. The ability of histamine to synergize LPS-induced COX-2 expression and prostanoid production underscores the potential role of this mast cell mediator to amplify infection-associated inflammatory responses. Since a physiological balance in the production of PGI_2_ and TXA_2_ by endothelial cells is critical for maintaining vascular integrity and controlling thrombosis, the histamine-induced shift of prostanoid equilibrium in favor of PGI_2_ production is noteworthy and supports its well-recognized vasodilatory and vasoprotective functions.

## Role of Mast Cells in Vascular Inflammation and Atherosclerosis

Mast cells alter the tissue microenvironment and modulate vascular inflammation, progression of AS, cardiac ischemia, and CVD (19). Mast cell progenitors reach the tissue through a chemokine-mediated mechanism and differentiate into mucosal or connective tissue phenotypes. Mucosal mast cells are characterized by the presence of only tryptase and chondroitin sulfate (proteoglycan), whereas connective tissue mast cells contain tryptase as well as chymase and heparin (proteoglycan) (20). Mast cells release vasoactive and angiogenic compounds and proinflammatory mediators, such as arachidonic acid metabolites (prostaglandin D_2_, leukotrienes), histamine, cytokines (e.g., IL-6 and IFNγ) and chemokines (e.g., eotaxin, MCP-1, and RANTES), platelet-activating factor (PAF), heparin, and proteolytic enzymes (21). These effector molecules are packaged in electron-dense secretory granules (mast cell granules, MCG). Endothelial cells can endocytose MCG *in vitro* (22) and *in vivo* (23). MCG can also promote human microvascular endothelial cell proliferation (24), LDL uptake by macrophages, and foam cell formation (21, 25).

Risk factors such as hypercholesterolemia, hyperglycemia, proinflammatory cytokines, oxidized LDL, reactive oxygen species, complement 5a, substance P, endothelin-1, and thrombin can activate mast cells (25). Mast cells express proangiogenic cytokine βFGF and participate in neovascularization and release histamine that increases vascular permeability and proinflammatory effects on endothelial cells (26–28). Clinical significance of mast cells is evident by their accumulation in the human arterial intima and adventitia where they contribute to atherosclerotic plaque development as well as its destabilization through their secretory products (21, 22, 29). Increased number of mast cells is noted in coronary arteries during spasm and in the rupture-prone shoulders of coronary atheromas (2). Although these findings implicate mast cells in vascular homeostasis, the mechanism by which they promote atherogenesis and CVD is not well understood.

The direct role of mast cells in AS is evident from studies by us and others, showing attenuation of AS progression in *ApoE^−/−^/Kit^W-sh/W-sh^* or *LDLr^−/−^/Kit^W-sh/W-sh^* mice (30–33). These mouse models are generated by crossing AS-prone *ApoE^−/−^* or *LDLr^−/−^* mice with mast cell-deficient *Kit^W-sh/W-sh^* mice. Using *ApoE^−/−^/Kit^W-sh/W-sh^* mice, we demonstrated that mast cell deficiency attenuates AS progression in *ApoE^−/−^* mice. Interestingly, the reduction in atheroma development in *ApoE^−/−^/Kit^W-sh/W-sh^* mice was associated with marked decreases in hepatic steatosis, reduced serum levels of cholesterol, LDL, and HDL, and decreased number of T-lymphocytes and macrophages in atheromas. Furthermore, *ApoE^−/−^/Kit^W-sh/W-sh^* mice presented significantly lower serum IL-6 and IL-10, with no changes in the levels of IL-2, IL-4, IL-17, IFNγ, and TNFα. Mast cell deficiency did not reduce serum levels of IFNγ in the *ApoE^−/−^* mice, suggesting that mast cell-derived IFNγ may not play a role in this mouse model of AS as opposed to the *LDLr^−/−^* mouse model (31, 34). Mast cell deficiency did not alter systemic production of TXA_2_ in *ApoE^−/−^* mice but led to significant reduction in the production of PGI_2_. Significantly lower expression of COX-2 mRNA in the aortic tissues of *ApoE^−/−^Kit^W-sh/W-sh^* mice compared with that of *ApoE^−/−^* mice suggests that mast cells regulate COX-2 expression. In this regard, the ability of histamine to induce COX-2 expression and PGI_2_ production in human endothelial cells and the markedly reduced expression of COX-2 mRNA in the aorta of histidine decarboxylase-null mouse (7) emphasize the importance of histamine in prostanoid homeostasis. Taken together, the marked reduction of hypercholesterolemia and vascular inflammation due to mast cell deficiency may attenuate AS progression in *ApoE^−/−^/Kit^W-sh/W-sh^* mice.

## Role of Histamine and H1 Receptor in Endothelial Cell Inflammatory Response

Histamine is a major secretory product of the mast cell that regulates vasodilation and bronchoconstriction (33, 35) and acts in combination with proteases to destabilize the plaque (35). Histamine induces smooth muscle cell migration and proliferation (36) and regulates intimal thickening (37). Histamine also regulates functions of monocytes and macrophages and eosinophils (38, 39), T-cells (40), neutrophils, and endothelial cells (41, 42). Histamine acts through a family of four distinct G-protein-coupled receptors (GPCR), namely, H1R, H2R, H3R, and H4R (43–45). Endothelial cells and smooth muscle cells highly express H1R, which facilitates histamine-mediated inflammatory and hypersensitivity responses (5–7, 33, 35, 36).

The role of histamine in AS and myocardial damage began to emerge only recently (46–48). Coronary arteries of patients with ischemic heart disease contain more mast cells and histamine than normal vessels (46), and patients with variant angina have elevated levels of histamine in their coronary circulation (49). Increased H1R mRNA expression has been reported in smooth muscle cells of intima/media in the atheroma (50). We found that histamine acting through H1R stimulates the expression of IL-6 and COX-2, with increased production of PGI_2_ by human endothelial cells. Interestingly, these effects are synergistically enhanced by LPS which suggests that mast cell products and bacterial agents act in concert to enhance vascular inflammation.

## Infection, Inflammation, and Atherosclerosis

Current literature strongly suggests a key role for infection-related immune activation in AS. *Chlamydia pneumoniae*, *Porphyromonas gingivalis*, *Aggregatibacter actinomycetemcomitans*, *Helicobacter pylori*, and cytomegalovirus have been shown to be present in plaques and to promote AS in animals (51–56). LPS, a cell wall glycolipid of Gram-negative bacteria, induces the production of proinflammatory cytokines and the expression of adhesion molecules on endothelial cells. Gram-positive bacterial cell wall components, peptidoglycan (PGN) and lipoteichoic acid (LTA), also induce endothelial cell activation (5, 6). LPS is detectable in apparently healthy individuals with subclinical or chronic infections such as periodontitis, sinusitis, bronchitis, or diverticulitis. Persistent LPS levels as low as 50 pg/mL may be a strong risk factor for AS, particularly among smokers. Our studies demonstrated that bacterial toxins and histamine synergistically enhance endothelial inflammatory response through overexpression of TLR2 and TLR4 (5–7).

Activation of endothelial cells by pathogen-associated molecular patterns (PAMPs) and/or molecules from damaged host tissues induce the expression of inflammatory cytokines and cell adhesion molecules (57–60). TLRs are innate immune receptors that recognize PAMPs (61). TLR4 is the signaling receptor for LPS or heat shock protein-60 (human and chlamydial) (62, 63). TLR2 recognizes Gram-positive bacterial, mycobacterial, and fungal cell wall components (64, 65).

Increasing evidence suggests that TLR2 and TLR4 modulate vascular inflammation and atherosclerotic disease (66, 67). Endothelial layers of atherosclerotic lesions express higher levels of TLR2 and TLR4 mRNA, and patients with loss-of-function TLR4 polymorphism have reduced risk of CVD (68). Mutations in TLR4 have been linked with a lower incidence of AS and other CVDs (69). Furthermore, TLR4 gene deletion reduces vascular inflammation and atherogenesis in *ApoE^−/−^* mouse (70). Anecdotally, it is believed that viral or bacterial infection increases the chances for “acute coronary syndrome.” Despite putative significance of TLR2 and TLR4 in AS (67–70), an association between their expression in the plaque and severity of atherosclerotic disease is not established. Our preliminary clinical study showed an association between overexpression of TLR2, TLR4, and COX-2 in the carotid artery plaque and the severity of widespread AS including the presence of peripheral artery disease (71). These observations underscore the clinical significance of an overactive TLR2/TLR4/COX-2 axis in CVD.

## The Synergy Between Mast Cell Histamine and Bacterial Toxins

The TLR-mediated endothelial response to bacterial pathogens may be modulated by mast cell mediators. Our studies show that histamine acting through H1R stimulates the expression of TLR2, TLR4, IL-6, COX-2, PGI_2_S, and PGE_2_S genes, leading to enhanced production of IL-6, PGE_2_, and PGI_2_ by HCAECs (5–7). We recently showed that LPS upregulates H1R gene and protein expression in HCAECs. We also noted that LPS-exposed HCAECs expressed threefold higher H1R than quiescent cells as assessed by H1R ligand binding. Furthermore, these cells were hyperresponsive to histamine as indicated by increased production of PGI_2_, PGE_2_, and IL-6. The LPS-induced hyperresponsiveness to histamine challenge was found to be mediated via H1R since it was abrogated by H1R antagonists (5–7, 72, 73). Thus, convergence of reciprocally upregulated H1R and TLR2/TLR4 functions was evident by the enhanced translocation of NF-κB and inflammatory response in endothelial cells treated with histamine in the presence of TLR ligands (74).

## Cigarette Smoke, Innate Immune System, and Atherosclerosis

Cigarette smoke is a major cause of cardiovascular morbidity and mortality (75, 76). CS predisposes the individual to aortic and peripheral AS leading to clinical atherosclerotic syndromes, including stable angina, acute coronary syndromes, sudden death, and stroke (75). CS impacts all phases of AS from endothelial dysfunction to acute clinical events, the latter being largely thrombotic (77–84).

TLR recognition is not restricted to exogenous microbial patterns. Putative endogenous ligands include fibronectin extra domain A fragment, heat shock proteins, and hyaluronan fragments. Endogenous ligands may be released during tissue damage and drive inflammation in the absence of infection. Therefore, a risk factor like CS can potentially promote inflammation via these ligands. Indeed, gaseous and particulate constituents of CS increase TLR2, 4, and 6 expression by gingival epithelial cells (85). It is worth mentioning that CS may deliver bioactive LPS to smokers which may contribute to bronchitis in smokers. However, these investigators did not detect differences in the circulating levels of LPS between non-smokers and smokers (86).

Cigarette smoke upregulates both TLR4 expression and the release of IL-8 and LTB4 in alveolar macrophages (87). CS also induces TLR4 and MMP4 expression in human small airway epithelial (SAE) cells and lung tissue (88). Furthermore, CS has been shown to activate mast cells to release preformed mediators such as histamine and to inhibit prostaglandin production (34).

It is noteworthy that CS not only induces COX-2 expression and its enzyme activity but also increases PGE_2_ and TXA_2_ release (89, 90). CS extract (CSE) and sera from smokers increase COX-2 expression in endothelial cells (91), and this change is associated with augmented angiogenesis in the atherosclerotic plaque. CS may exert proinflammatory effects in a PGE_2_-dependent manner while TXA_2_ may mediate its proatherogenic effect via platelet activation, vasoconstriction, and angiogenesis (91).

## The “H1R–TLR–COX2 Axis” – An Integrated Approach to Study the Immunomodulatory Effects of CS and Infection on Atherogenesis

**Figure 1:**
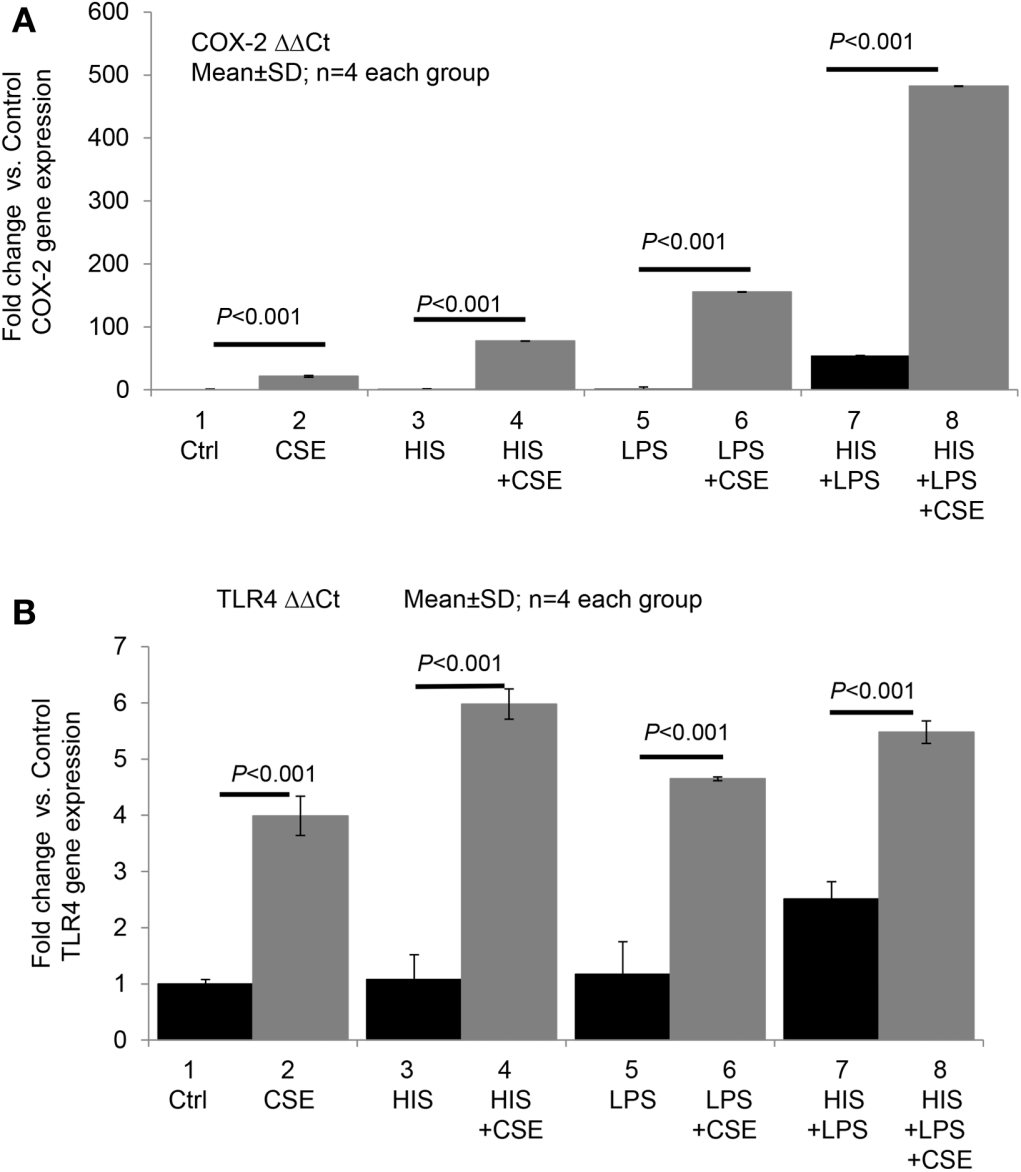
**Cigarette smoke extract directly and synergistically with histamine or LPS enhances the expression of COX-2 and TLR4 in human endothelial cells**. Human umbilical vein endothelial cells (HUVECs) were grown to confluence and incubated with (1) medium (control), (2) cigarette smoke extract (CSE) (final concentration equivalent to 100 nM nicotine), (3) histamine (HIS, 10 μM), (4) HIS + CSE, (5) LPS, 100 ng/mL, (6) LPS + CSE, (7) HIS + LPS, and (8) HIS + LPS + CSE for 24 h. After the incubation, total RNA was extracted and used for RT-qPCR analyses of gene expressions of COX-2 **(A)** and TLR4 **(B)**. Data presented are mean ± SD of quadruplicate experiments. CS increased COX2 gene expression significantly compared to control, HIS, LPS, or HIS + LPS groups (*P* < 0.001). HIS + LPS (6) increased COX2 expression compared to control, HIS, or LPS groups (*P* < 0.001). CS increased TLR4 gene expression significantly compared to control, HIS, LPS, or HIS + LPS groups (*P* < 0.001). HIS + LPS (6) increased TLR4 expression compared to control, HIS, or LPS groups (*P* < 0.001).

We recently conducted a pilot study to test whether CSE directly or synergistically modulates the effect of histamine or LPS on COX-2 and TLR4 expression in endothelial cells. Briefly, human umbilical vein endothelial cells (HUVECs) were incubated with medium (control), histamine, or LPS alone and in combination with CSE for 24 h (see Figure 1). As shown in Figure 1A, CSE significantly increased the basal as well as histamine- and LPS-induced COX-2 gene expression with varying magnitudes of synergy. Similarly, CSE significantly enhanced the expression of TLR4 mRNA in quiescent and in histamine- and LPS-treated cells (Figure 1B). These results indicate that CS can upregulate COX-2 and TLR4 pathways.

**Figure 2:**
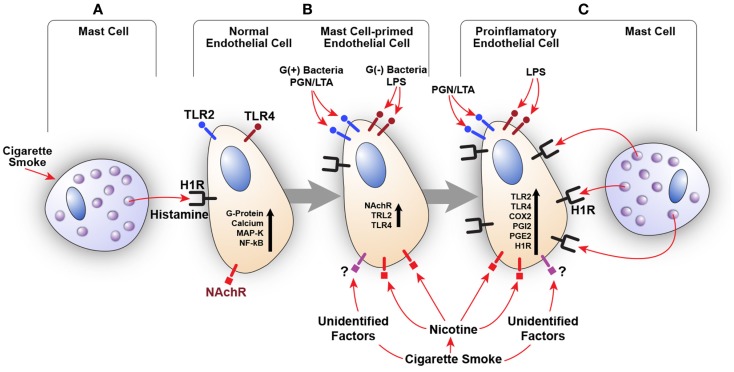
**Amplification of endothelial inflammatory response by cigarette smoke (CS) and bacterial products via innate immune upregulation**. **(A)** Histamine secreted by the mast cell stimulates H1R on endothelial cells. CS may also increase histamine release. **(B)** H1R-mediated endothelial cell activation leads to increased expression of TLR2/TLR4 and nicotinic acetylcholine receptors (NAChR). This cross talk programs endothelial cells to become hyperresponsive to the TLR2/TLR4 ligands (PGN, LTA, and LPS) and CS leading to enhanced inflammatory response. **(C)** Increased TLR2/TLR4 signaling also increases H1R expression. Collective stimulation of newly expressed TLR2/TLR4 and H1R leads to robust proinflammatory changes in the endothelium and persistent vascular inflammation.

We also examined the effect of histamine on the expression of nicotinic acetylcholine receptor α1 (NAChRα1) mRNA in HUVECs. Histamine upregulated NAChRα1 gene expression, which was enhanced by LPS (data not shown). These findings suggest that histamine increases endothelial cell sensitivity to nicotine and CS through overexpression of NAChRα1. The involvement of other nicotinic acetylcholine receptors as well as receptors that recognize unidentified factors in CSE remains to be determined. Based on these preliminary results, our published work (5–7, 72–75, 77–83) and a recent clinical study correlating TLR2, TLR4, and COX-2 overexpression in carotid artery plaques with the severity of disease (71), we postulate that constituents of CS, acting in concert with bacterial toxins and mast cell products, may amplify inflammatory response by engaging the H1R-TLR2/TLR4-COX2 axis (Figure 2).

## Conclusion

Mast cells and TLRs are constituents of the innate immune system, and they synergistically enhance proatherogenic inflammatory response in endothelial cells. Our published work shows that histamine acting via H1R, and in conjunction with TLR2 or TLR4 ligands, synergistically amplifies the expression of COX-2 and IL-6 in endothelial cells with resultant overproduction of PGI_2_, PGE_2_, and IL-6. Preliminary data also show that CS extract enhances histamine- and LPS-induced endothelial expression of COX-2 and TLR4 in a synergistic manner, and histamine induces NAChRα1 expression. We propose that whole CS as well as its constituents such as nicotine modulate innate immune system and amplify infection-mediated proatherogenic inflammatory responses to promote the onset and progression of AS.

## Conflict of Interest Statement

The views expressed in this article are those of the authors and do not necessarily reflect the position or policy of the Department of Veterans Affairs or the United States Government. All authors have reviewed and approved the manuscript. All authors have read the journal’s policy on conflicts of interest. None of the authors have any conflict of interest to declare regarding the contents of this paper.
